# Clinical role of CYP1B1 gene polymorphism in prediction of postoperative chemotherapy efficacy in NSCLC based on individualized health model

**DOI:** 10.1515/biol-2022-0705

**Published:** 2023-10-16

**Authors:** Bo Liu, Shaofeng Zhang, Chunyan Liu, Xia Han

**Affiliations:** Xingtai People’s Hospital, Xingtai 054001, Hebei, China; Department of Chest Surgery, Xingtai People’s Hospital, Xingtai 054001, Hebei, China; Department of Thoracic Surgery, Xingtai People’s Hospital, Xingtai 054001, Hebei, China

**Keywords:** individualized health model, CYP1B1 gene polymorphism, NSCLC, postoperative chemotherapy efficacy, SPSS algorithm

## Abstract

Non-small cell lung cancer (NSCLC) is one of the most common cancers worldwide, and chemotherapy is one of its main treatment methods. However, there are significant differences in patients’ reactions to chemotherapy, leading to unsatisfactory treatment outcomes. Therefore, identifying relevant factors that affect the efficacy of chemotherapy can help doctors better develop personalized treatment plans, improve the treatment effectiveness, and quality of life of patients. This article aims to understand the specific clinical role of CYP1B1 gene in NSCLC. Therefore, based on the individualized health model of CYP1B1 gene polymorphism, this article analyzes the prediction of postoperative chemotherapy efficacy for NSCLC. Through a study on the control variables of postoperative recovery of stage III NSCLC in a hospital, according to the findings of this study, 14 of the 32 patients in the EGFR mutation-positive group relapsed. In the EGFR-negative group, 13 of the 36 patients relapsed. It can be considered that CYP1B1 gene polymorphism has a good curative effect in postoperative chemotherapy of NSCLC, and it can effectively control the recurrence rate of cancer.

## 1 Introduction

Non-small cell lung cancer (NSCLC) accounts for 75–80% of all lung cancer patients, of which 75–80% are at an advanced stage when they are diagnosed, and only 20–30% of cases have the opportunity for surgery. For this part of the population, even if radical surgical resection is performed, 70% of patients will eventually fail due to recurrence and distant metastasis. In theory, postoperative adjuvant chemotherapy could eliminate these tiny remnants and improve survival.

With the in-depth study of CYP1B1 gene polymorphism in individualized healthy models, it is believed that the CYP1B1 gene of the cytochrome p450 family is related to the susceptibility rate of NSCLC. The CYP1B1 gene is a gene encoding an enzyme that is involved in processes such as cancer cell metastasis, invasion, and drug resistance, and is associated with oxidative stress. The polymorphism of this gene has been proven to be associated with the risk of multiple tumors and treatment responses. Therefore, its clinical role in CYP1B1 gene polymorphism in predicting the efficacy of postoperative chemotherapy in NSCLC is very necessary. Analyzing the correlation between CYP1B1 gene polymorphism and efficacy can provide important basis for the design of personalized treatment plans for NSCLC patients, thereby improving treatment effectiveness and reducing side effects. This has important clinical significance for achieving precision medicine and optimizing tumor treatment.

For the study of NSCLC, this article has the following innovations. (1) This article analyzes the dynamics of CYP1B1 gene based on an individualized health model, which highlights its convenient mechanism in NSCLC. (2) For the prediction of the efficacy of postoperative chemotherapy for NSCLC, this article selected three different postoperative populations for experiments. This ensures that the role of CYP1B1 gene dynamics on the global burial bed can be distinguished.

## 2 Related work

At present, it is believed that CYP1B1 gene polymorphism plays an important role in the efficacy of postoperative chemotherapy for NSCLC. There are many studies on CYP1B1 gene polymorphism. CecchinE found that there was no significant difference between the CYP1B1*3 case group and the control group [1]. Fassad used polymerase chain reaction (PCR)/restriction fragment length polymorphism to screen patients and 100 healthy subjects (controls) for two mutations in the CYP1B1 gene and one mutation in the MYOC gene. Phenotypic features associated with disease severity were compared [2]. Ivanoshchuk performed direct automated Sanger sequencing of CYP1B1 gene exons and adjacent splice sites in 28 individuals with poly comb group phenotype from the Western Siberia region [3]. To understand thyroid disease, especially Hashimoto’s thyroiditis, Kowalczyk found that it occurs at a significantly higher rate in patients with polycystic ovary syndrome (PCOS) than in the general population. They are about 27 and 8%, respectively. The aforementioned common etiologies associated with fertility problems in hashimoto thyroiditis and PCOS require further investigation [4]. However, it can be found that the existing research is more about the analysis of the principle and potential use of CYP1B1 mutation and its dynamics, and there is no analysis of the postoperative efficacy of NSCLC. At present, the research on CYPIBI gene abroad mainly focuses on breast cancer, endometrial cancer, and glaucoma. So far, there are few studies on CYPIBI gene polymorphism and hereditary susceptibility to lung cancer, and its protein expression is mainly studied. However, there is no research report on CYPIBI gene polymorphism and genetic susceptibility to lung cancer in China.

## 3 CYP1B1 gene polymorphism

### 3.1 Introduction to CYP1B1

CYPIBI is an important cytochrome oxidase located on chromosome 2 2p21-p22. It has three exons and two introns, and the open reading frame starts from the second exon, totaling 1,629 bp. It encodes an enzymatic protein consisting of 543 amino acids. In addition to participating in drug metabolism, CYP1B1 also participates in the metabolism of endogenous hormones, such as estrogen and testosterone, and plays an important role in the metabolism of some carcinogen. Defects or mutations in CYP1B1 may lead to the occurrence of some diseases, such as cataracts, glaucoma, tumors, etc. It has been found that CYPIBI has six polymorphic sites, four of which lead to amino acid changes, and its allele frequencies have ethnic differences. CYP1B1 is widely present in extrahepatic tissues, such as lung, breast, uterus, central nervous system, etc. It can oxidatively metabolize many pro-carcinogens (e.g., catalyze the activation of polycyclic aromatic hydrocarbons and aromatic amines) and body hormones (e.g., mediate the lightening of 17-estradiol to form 4-catechol estrogens). Compared with CYP1A1, 1A2, 3A3, and 3A4, CYP1B1 had the highest catalyzing activity of estradiol 4-light, which is the main enzyme of 4-light metabolism. Some studies suggest that CYP1B1 is specifically expressed in tumor tissues. Therefore, CYP1B1 is not only an important enzyme in the bioactivation of environmental carcinogens, but also in the regulation of steroid hormones. The generation of some toxic steroid hormone metabolites is also of great significance. In cells, CYP1B1 can metabolize estrogen and convert it into secondary metabolites, thereby affecting the biological effects of estrogen.

Normally, activation/deactivation of pro-carcinogens is in a state of homeostasis [5,6]. When one or both of these two processes are changed, it leads to some activated intermediates such as epoxides, semi-alkaloids, etc., which bind to DNA bases and become final carcinogens. For example, when AHH enzyme (aromatic hydrocarbon lightenase) activity increases, it disrupts this balance [7,8]. If the GSTMI and other genes in the reaction are deleted, it will not have the activity of the enzyme, and the ability to prevent the formation of DNA adducts will be lost. It prevents the intermediate products from being excreted from the body, and combines with DNA to form carcinogens, which can easily lead to normal cells becoming cancerous. It is currently believed that foreign pro-carcinogens enter the body and are activated into metabolically active intermediates under the action of phase I metabolic enzymes such as cytochrome P450. It then enters the phase reaction and is excreted in combination with phase II metabolizing enzymes such as glutathione-S-transferase (GSTS). This process is deactivation or inactivation. Members of the GSTS family use different thiol auxiliary substrates such as glutathione to bind pre-carcinogenic metabolites to them, making them easier to excrete from the body.

**Figure 1:**
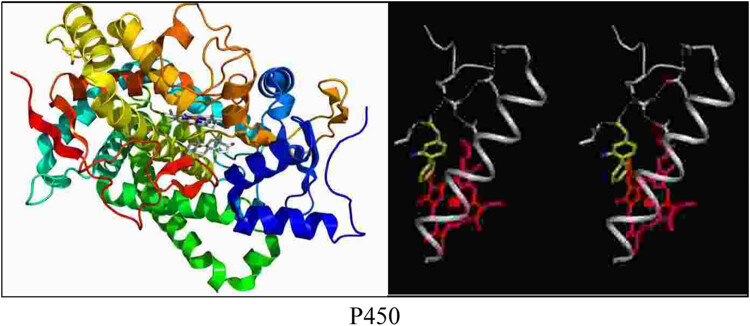
Cytochrome p450.

**Figure 2:**
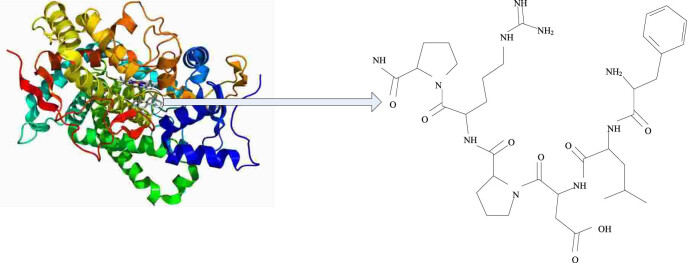
Structure of CYP1B1.

Cytochrome P450 (referred to as CYP450) represents a large family of autooxidizable heme proteins, belonging to a class of monooxygenases. Due to the wide variety and subtypes of metabolic substances involved in CYP450, it has broad clinical application value. For example, many drugs need to be metabolized through the CYP450 enzyme in order to achieve therapeutic effects. It is named for its specific absorption peak at 450 nm. It is involved in the metabolism of endogenous substances and exogenous substances including drugs and environmental compounds. As an extrahepatic metabolic enzyme, CYP1B1, on the one hand, forms DNA adducts in the metabolic activation environment of polycyclic aromatic hydrocarbons, heterocyclic amines, aromatic amines, etc., leading to the formation of tumors. On the other hand, it produces semiquinones and quinones in 17β-E2 in metabolically oxidized tissues, which have toxic effects on DNA and induce carcinogenesis. CYP1B1 is a relatively conservative gene sequence, its gene is located on chromosome 2p21-22, and its full length is 12 kb. In the long-term human evolution, there are mainly four loci affecting enzyme activity, namely R48G, A119S, L432V, and N453S. Among them, R48G, A119S, and L432V site mutations can increase the catalytic activity of CYP1B1; however, N453S may have reduced activity in the metabolism of pro-carcinogens, such as polycyclic aromatic hydrocarbons. Many studies have shown that CYP1B1 gene locus polymorphism can increase the susceptibility of hormone-dependent tumors such as breast cancer, endometrial cancer, and prostate cancer. However, these tumor susceptibility may be related to ethnicity and geography [9,10]. Therefore, it is presumed that CYP1B1 key locus gene polymorphisms can increase the susceptibility of NSCLC, which may explain individual differences. Geographical differences can lead to sensitivity results in NSCLC. The cytochrome p450 and CYP1B1 genes and their structures are shown in Figures 1 and 2.

### 3.2 CYP1B1 oncogenic pathway

#### 3.2.1 Formation of intermediates such as epoxides or diol epoxides

CYP1A1 and CYP1B1 mainly activate PAHs to form epoxide intermediates, which then form PAH-DNA adducts. If the mutation occurs at key DNA sites, it will lead to the loss of normal regulation of cells, leading to tumorigenesis [11,12]. CYP1A1 and CYP1B1 are important members of the CYP450 subtype and are considered key enzymes in the polyaromatic hydrocarbons (PAH) metabolism process. The expression of these two enzymes has been found in both domestic animals and normal human livers. When PAH enters the body, it will be metabolized by CYP450 enzyme to form active metabolite, such as epoxides. These metabolites can combine with nucleic acid bases in DNA to form adducts. In environments exposed to PAH, this phenomenon may lead to DNA damage and mutations, thereby increasing the risk of cancer. In a study of mouse cutaneous papillomas, PAH-DNA apurine adducts were found to be associated with levels of tumorigenic H-ras mutations. Potentially carcinogenic 7,12-xylene anthracene and dibenzopyrene preferentially form adenylate depurine adducts and induce A>T base transitions at codon 61. In contrast, the depurine adduct of guanylate formed by benzopyrene and the G>T base transition formed at codon 13 were twice as high as those of 7,12-xylene anthracene and dibenzopyrene [13,14]. Therefore, the carcinogenic effects of benzopyrene are more sensitive. In addition, AFB1-8,9-double bond can be epoxidized under the action of CYP1A1 and CYP2E1. The formed aflatoxin B1 exo-8,9-epoxide (AFBO) is an electrophilic compound. It forms adducts with intracellular DNA and proteins, directly causing DNA damage, and finally causing cell canceration. AFBO is a highly electrophilic compound that can form covalent adducts with DNA, RNA, and proteins within cells. The formation of these adducts can lead to the formation of DNA attachments and DNA damage, thereby increasing the risk of cancer occurrence. In addition, AFBO can also inhibit cell apoptosis, enhance cell proliferation, and promote tumor development.

#### 3.2.2 Formation of activated semiquinones and quinones

**Figure 3:**
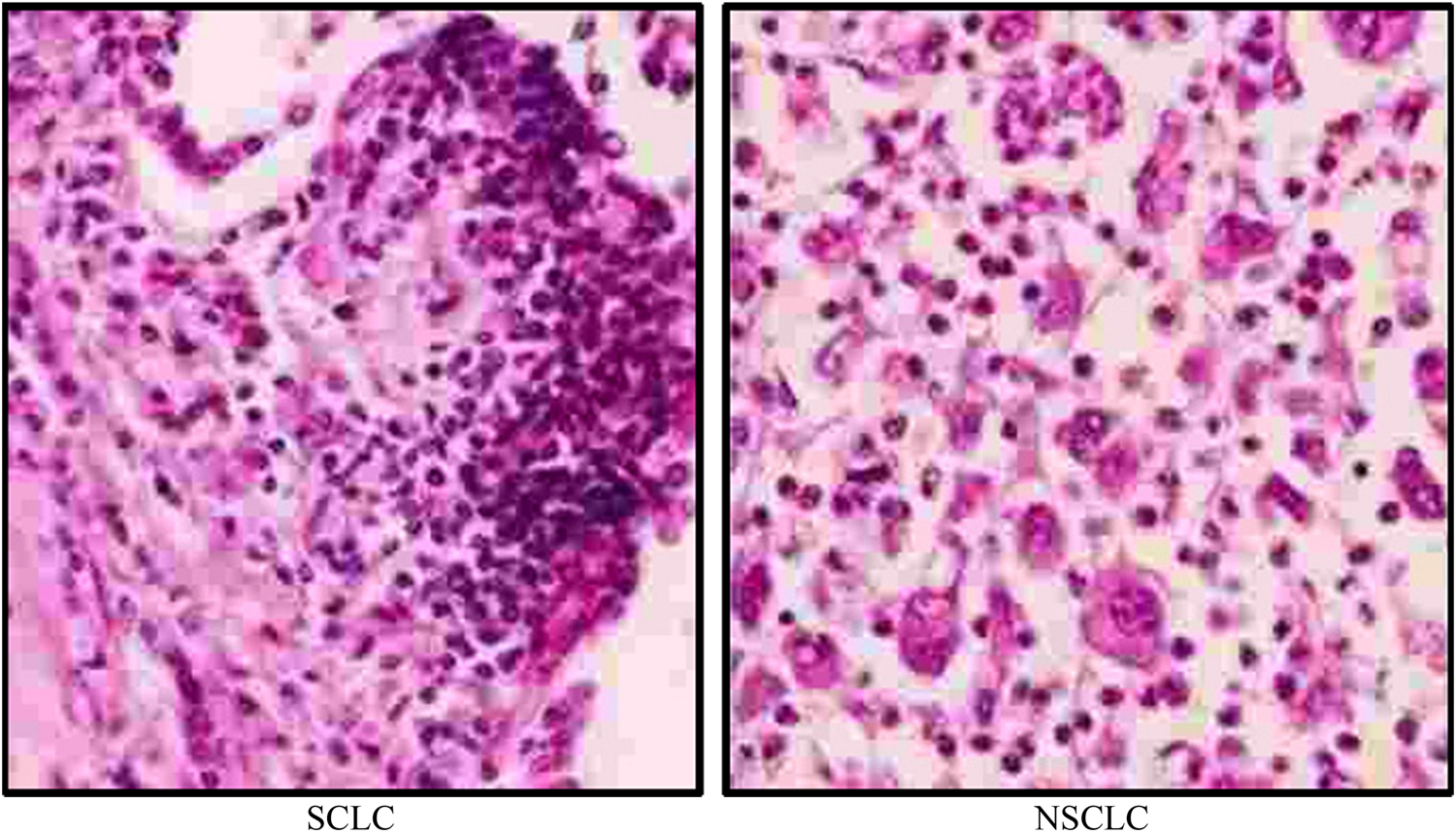
SCLC and NSCLC.

Estrogen (estrone E1, estradiol E2), dopamine, and benzene all contain a benzene ring structure, which can be oxidized to semiquinones and quinones. Under the catalysis of CYP1A1 and CYP1B1, estrogens (E1, E2) are converted into catechol estrogens (2-OHE1(E2), 4-OHE1(E2)) and then gradually oxidized to form semiquinones and quinones (E1(E2)-2,3-Q, E1(E2)-3,4-Q). Among them, 4-OHE1(E2) exerts oncogenic effects in all animal models, while 2-OHE1 (E2) is not a carcinogenic or only forms borderline tumors [15]. In addition, benzene can also form quinones and semiquinones under the catalysis of CYP2E1. Carcinogens metabolized in the body react with DNA to form two types of DNA adducts. Among them, less than 1% of the DNA adducts are stable adducts, i.e., they are not dissociated unless during DNA repair. In addition, more than 99% are depurinated adducts. It can be separated after binding to DNA, making DNA appear apurine sites. The mechanism is that the electrophilic reaction of carcinogenic DNA adducts occurs on adenine (Ade) and guanine (Gua) of the outer ring amino group. When the adduct binds to N3, adenylate at N7, and guanylate at N7, the stability of the glycosidic bond decreases, forming DNA depurine adducts. At the same time, there is no purine site in the DNA, which makes the DNA site mutated and induces canceration. Its mutagenic 4-OHE1(E2) and E1(E2)-3,4-Q can both form depurine adducts with DNA. The reaction of E1(E2)-3,4-Q with DNA occurred at N3Ade and N7Gua. Studies have shown that after injection of E2-3,4-Q into the mammary glands of female ACI mice, the levels of N3Ade and N7Gua adducts are approximately the same, and it detected mainly H-ras gene mutation (AT/GC). The rate of depurination of its N3Ade adduct occurs instantaneously, whereas depurination of the N7Gua site requires a half-life of several hours. The mutation rate of A base in H-ras gene is high, while a small part is G site mutation, which may be due to the faster depurination rate of N3Ade than N7Gua adducts. In addition, both estrogen metabolites and their depurinated DNA adducts were detected in human urine. Therefore, this adduct can serve as an important biomarker for tumorigenesis risk, as shown in Figure 3.

### 3.3 NSCLC

**Figure 4:**
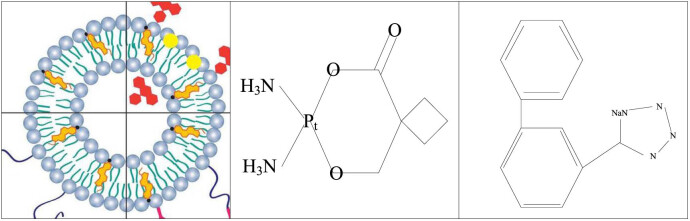
Structural diagram of the third-generation chemotherapeutic drugs.

The difference between small cell lung cancer (SCLC) and NSCLC is that SCLC has a higher degree of malignancy and requires chemotherapy to treat the disease, but the disease may not be completely cured. SCLC is a highly malignant lung cancer that accounts for approximately 10–15% of all lung cancer cases. SCLC cells have a relatively small morphology, high ratio of nucleus to cytoplasm, active nuclear division, fast growth rate, and are prone to invasion of surrounding tissues and distant metastasis. Therefore, SCLC often enters an advanced stage when detected and requires urgent treatment. NSCLC has a low degree of malignancy, and the disease can be treated by surgery, chemotherapy, radiotherapy, and targeted drugs. SCLC and NSCLC are shown in Figure 3. The so-called first-generation chemotherapy drugs for NSCLC refer to drugs with a single-drug effective rate of about 5%, such as doxorubicin (ADM), vincristine, and fluoropyrimidine (5-FU). Second-generation drugs refer to drugs that appeared after the 1970s and have a single-drug efficacy rate of about 10%, such as DDP, CBP, VDS, and IFO. The third-generation chemotherapeutic drugs refer to new drugs that appeared after the 1990s, and the single-drug efficacy rate exceeds 15%, such as NVB, Gemzar, Taxol, and Docitaxol. The structural diagram of these three generations of chemotherapy drugs is shown in Figure 4.

According to the world’s major research and exploration on the efficacy of adjuvant chemotherapy after radical resection of NSCLC in the past 20 years, with the emergence and clinical promotion of the second and third generation chemotherapy drugs, the effectiveness of adjuvant chemotherapy and the compliance of patients with chemotherapy regimens have been confirmed and improved, respectively, future clinical trials should focus on the clinical practice of a new generation of chemotherapy drugs. Adjuvant chemotherapy refers to the chemotherapy method used to eliminate small residual tumors and prevent tumor recurrence and metastasis after surgical resection of malignant tumors such as NSCLC. It compensates for the shortcomings of surgical resection in controlling local lesions and treating systemic lesions. It provides stronger evidence and guidelines for the clinical use of new drugs. In addition, since 2000, many scholars and research institutions have paid attention to the evaluation of the efficacy of adjuvant chemotherapy after radical resection of stage I NSCLC. This is certainly helpful for deepening our understanding of adjuvant preventive measures. However, it should not ignore the exploration of patients with locally advanced NSCLC as the trial population. Only by comprehensively examining the entire disease population can this article gain a better understanding of the relationship between disease and interventions, so that decisions that are most beneficial to patients in clinical practice can be made.

NSCLC refers to a type of malignant tumor that originates from lung tissue and belongs to the category of lung cancer. NSCLC accounts for approximately 85% of all lung cancers. Common types include adenocarcinoma, squamous cell carcinoma, and large cell carcinoma. The early symptoms of NSCLC are not obvious, while the late symptoms include persistent cough, expectoration, dyspnea, chest pain, weight loss, etc. The treatment goals of NSCLC are different according to the stage of NSCLC. For different patients, the goal of this article is cure. In contrast, for stage IV patients, the goal of palliative treatment is to relieve symptoms and prolong survival time. To achieve cure, the preferred intervention is radical surgery, including lobectomy and unilateral pneumonectomy. It is also combined with mediastinal lymph node dissection. Distant recurrence is the leading cause of death in patients with NSCLC within 5 years after radical resection, because even the tumor appears to be confined to the lung. However, some hard-to-find micrometastases are still the cause of distant recurrence. In this regard, many researchers try to eliminate the residual micrometastases after surgery by adjuvant chemotherapy after surgery, so as to achieve the purpose of reducing local recurrence and reducing distant metastasis. This article also reviews the research and attempts in adjuvant chemotherapy after radical resection of NSCLC in the past 20 years since 1992 in chronological order.

Cyclophosphamide, adriamycin, prednisone (CAP) adjuvant chemotherapy refers to a chemotherapy regimen used to treat NSCLC. CAP regimen consists of cyclophosphamide, adriamycin, and cisplatin, which is one of the most commonly used chemotherapy schemes in clinical practice. Adjuvant chemotherapy with CAP regimen is not expected to use for patients with stage I NSCLC after radical resection. This article attributes the low efficacy of the adjuvant chemotherapy regimen for CAP indicated in the conclusions to poor patient compliance with the chemotherapy regimen and the poor efficacy of the chemotherapy regimen itself. Compared with a similar study conducted in Finland, the negative results of this study suggest that new adjuvant chemotherapy regimens that are more effective, have fewer toxic side effects, and have higher patient compliance that are needed in the future.

### 3.4 SPSS algorithm

Assuming an algorithm capable of estimating the aggregate demand over a fairly long period *T*, total demand can be estimated based on the user’s plan or forecasted based on historical records. So this assumption is reasonable, let

(1) \[L={\max }_{t}({d}_{t}).]\]

**Figure 5:**
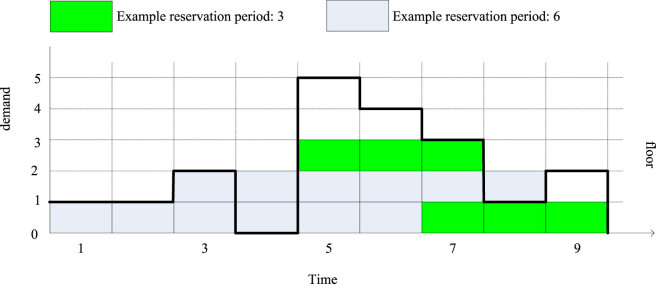
Demand curve and tier-wise reservation method with three reserved instance periods.

It is the peak demand, then the demand *d*_*t*_ is divided into *L* tiers. The definition index \[{d}_{t}^{l}]\] represents whether the *l*th layer has demand at time *t*, i.e., if *d*_*t*_ ≥ 1, then \[{d}_{t}^{l}]\] = 1, otherwise it is 0. For example, Figure 5 depicts a demand curve.

(2) \[\{{\tau }_{0}\right,{\tau }_{1},{\tau }_{2},{\tau }_{3}\}=\{1,3,6\left,10\},]\]

(3) \[\{{c}_{0}\right,{c}_{1},{c}_{2},c\}=\{1,2.5,3.8\left,6\}.]\]

\[{d}_{3}^{4}]\] = 0 because Tier 4 has no demand in billing cycle 3, and \[{d}_{5}^{5}]\] = 1, because Tier 5 has demand in billing cycle 5. In fact, each \[{d}_{t}^{l}]\] corresponds to a small rectangular shaded block. \[{d}_{t}^{l}]\] = 1 if the corresponding rectangle is below the curve, otherwise 0, obviously

(4) \[\mathop{\sum }\limits_{l=1}^{{d}_{t}}{d}_{t}^{l}={d}_{t}.]\]

A demand curve is said to be convex if

(5) \[\forall {t}_{0}\left\in [1\left,T].]\]

No other

(6) \[{t}_{1}\left\in [1\left,T],{t}_{2}\left\in [1\left,T].]\]

Let

(7) \[{t}_{1}\lt {t}_{0}\lt {t}_{2},]\]

(8) \[\mathop{\sum }\limits_{l=1}^{L}{d}_{{t}_{1}}^{l}\gt \mathop{\sum }\limits_{l=1}^{L}{d}_{{t}_{0}}^{l},]\]

(9) \[\mathop{\sum }\limits_{l=1}^{L}{d}_{{t}_{2}}^{l}\gt \mathop{\sum }\limits_{l=1}^{L}{d}_{{t}_{0}}^{l}.]\]

For example, in Figure 5, the demand curve at times 1–3 is convex. But the demand curve at times 4–9 is not convex because \[{\sum }_{l=1}^{L}{d}_{8}^{l}]\] is smaller than its neighbors.

\[{y}_{{ljt}}]\] is 1 if the request is served by a non- \[{\tau }_{0}]\] instance \[{\tau }_{j}]\] , otherwise 0. For example, in layer 2 of Figure 5, *y*_223_ = 1 because \[{\tau }_{2}]\] is reserved at time 3. *y*_209_ = 1 because a real-time instance \[{\tau }_{0}]\] is activated at time 9. All the rest \[{y}_{2{jt}}]\] = 0, the problem can be formalized as a mathematical programming 1 from a hierarchical perspective.

(10) \[\min \mathop{\sum }\limits_{l=1}^{L}\mathop{\sum }\limits_{t=1}^{T}\mathop{\sum }\limits_{j=0}^{J}{y}_{{ljt}}{c}_{j},]\]

(11) \[{\mathrm{s}}.{\mathrm{t}}.\mathop{\sum }\limits_{j=0}^{J}\left(\mathop{\sum }\limits_{t=1}^{T}\mathop{\sum }\limits_{j=0}^{J}({y}_{{ljt}}+{y}_{{\mathrm{lot}}})\right)\ge {d}_{t}\hspace{1em}t=1,2,\ldots ,T,]\]

(12) \[{y}_{{ljt}}\in \{0.1\},l=0,1,\ldots ,L{\mathrm{;}}j=0,1,\ldots ,J{\mathrm{;}}t=1,2,\ldots ,T.]\]

Because as long as \[{\tau }_{j}]\] is reserved, \[{c}_{j}]\] will be charged, so the objective function is the total cost. In constraint (2-1),

(13) \[\mathop{\sum }\limits_{j=1}^{J}\mathop{\sum }\limits_{i=t-{\tau }_{j}+1}^{t}{y}_{{lji}}.]\]

It is the number of reserved instances that are still valid at level *l* up to time *t*. \[{y}_{{\mathrm{lot}}}]\] is the number of real-time instances configured by layer *l* at time *t*. After calculating the sum of the instances of each layer. This number should not be less than the requirement \[{d}_{t}]\] .

Now, consider the following plan 0-1ILPForLevel corresponding to a single level:

(14) \[\min \mathop{\sum }\limits_{t=1}^{T}\mathop{\sum }\limits_{j=0}^{J}{y}_{{ljt}}{c}_{j}\left(01{\mathrm{ILPForLevel}}),]\]

(15) \[{\mathrm{s}}.{\mathrm{t}}.\mathop{\sum }\limits_{j=1}^{J}\mathop{\sum }\limits_{i=t-\tau +1}^{t}({{y}_{{ljt}}+y}_{{\mathrm{lot}}})\ge {d}_{t}^{l},\hspace{1em}t=1,2,\ldots ,T,]\]

(16) \[{y}_{{ljt}}\left\in \left\{0.1\right\},\hspace{1em}j=0,1,\ldots ,J{\mathrm{;}}\hspace{1em}t=1,2,\ldots ,T,]\]

(17) \[f(y)=\mathop{\sum }\limits_{t=1}^{T}\mathop{\sum }\limits_{j=0}^{J}{y}_{{ljt}}{c}_{j}.]\]

Let *y*∗ be the optimal solution of planning 0-1ILPForLevel, and *y* be any feasible solution of planning 0-1ILPForLevel, then *f*(*y*) ≥ *f*(*y*∗), so

(18) \[\mathop{\sum }\limits_{l=1}^{L}f(y)\ge \mathop{\sum }\limits_{l=1}^{L}f({y}^{* }).]\]

Then

(19) \[\min \mathop{\sum }\limits_{l=1}^{L}f(y)\ge \min \mathop{\sum }\limits_{l=1}^{L}f({y}^{* })=\mathop{\sum }\limits_{l=1}^{L}f({y}^{* }).]\]

## 4 Materials and methods for efficacy prediction

### 4.1 Specimen source and related clinicopathological data

#### 4.1.1 Standard constrain

(1) Postoperative pathological diagnosis of NSCLC patients. (2) Patients who underwent radical lung cancer resection with negative margins and received 3–4 courses of gemcitabine cisplatin (GP) chemotherapy after surgery. (3) Patients with clear diagnosis, over 18 years of age, and normal cognitive ability. (4) Patients with physical condition score of 0–1 (ECOG standard). (5) Patients with a survival period of more than 6 months. (6) Patients with good bone marrow, kidney, liver, and heart and lung functions meet the following criteria, as shown in Table 1.

**Table 1:** Included indicators and scope of indicators

White blood cell count	≥4.0 × 10^9^/L and ≤10.0 × 10^9^/L
Neutrophil count	≥2.0 × 10^9^/L
Platelet count	≥100 × 10^9^/L
Hemoglobin	≥95 g/L
AST (GOT)	≤100 U/L
ALT (GPT)	≤125 U/L
Serum total bilirubin	≤45 μmol/L
Serum creatinine	≤115 μmol/L
LVEF (Echocardiography/ECT: left ventricular ejection fraction)	≥55%


**Informed consent:** Informed consent has been obtained from all individuals included in this study.
**Ethical approval:** The research related to human use has been complied with all the relevant national regulations, institutional policies and in accordance with the tenets of the Helsinki Declaration, and has been approved by the authors’ institutional review board or equivalent committee.

#### 4.1.2 Exclusion criteria

(1) Patients with incomplete resection of the primary tumor and/or positive margins. (2) Patients with definite interstitial pneumonia or pulmonary fibrosis on chest imaging examination. (3) Patients with severe infection, abnormal cardiac function (heart failure or abnormality requiring treatment on electrocardiogram) or severe liver and kidney dysfunction. (4) Patients with active gastric and duodenal ulcers. (5) Patients with active repeat cancer (excluding intraepithelial cancer and repeat cancer that has not recurred for more than 5 years). (6) Patients whose address or contact information has been changed and follow-up cannot be obtained.

#### 4.1.3 Basic clinicopathological characteristics of selected cases

According to the above criteria, 68 patients with NSCLC who underwent radical lung cancer resection in the Thoracic Surgery Department of a First Hospital from October 2008 to February 2012 and received GP regimen adjuvant chemotherapy after surgery were selected. All specimens were obtained from paraffin sections of surgically resected specimens collected by thoracic surgery of a first hospital (tumor tissue accounted for more than 70%). There were 47 men and 21 women among them, with an age range of 30–73 years and a median age of 51.5 years. Squamous cell carcinoma (32 instances), adenocarcinoma (31 cases), and adenosquamous carcinoma (five cases) were the pathological forms.

#### 4.1.4 Observation method and evaluation index

(1) Follow-up: the starting point of follow-up is the first day of the operation date, and the deadline is March 1, 2013. The methods include telephone follow-up and outpatient review records. The follow-up contents included general condition, clinical symptoms, and imaging examinations. (2) Evaluation indicators are disease-free survival rate (DiseaseFreeSurvivalrate, DFS) and median progression-free survival time (ProgressFreeSurvival, PFS). Disease-free survival refers to patients receiving a certain treatment. After several years of follow-up, the proportion of patients without recurrence accounted for the proportion of patients who were followed up for *n* years (*n*-year disease-free survival rate = patients who were followed up for *n* years without recurrence/patients who were followed up for *n* years). Progression-free survival refers to the time from the start of treatment to the observation of disease progression or death from any cause in patients with tumor disease.

### 4.2 Main PCR reagents and methods

**Figure 6:**
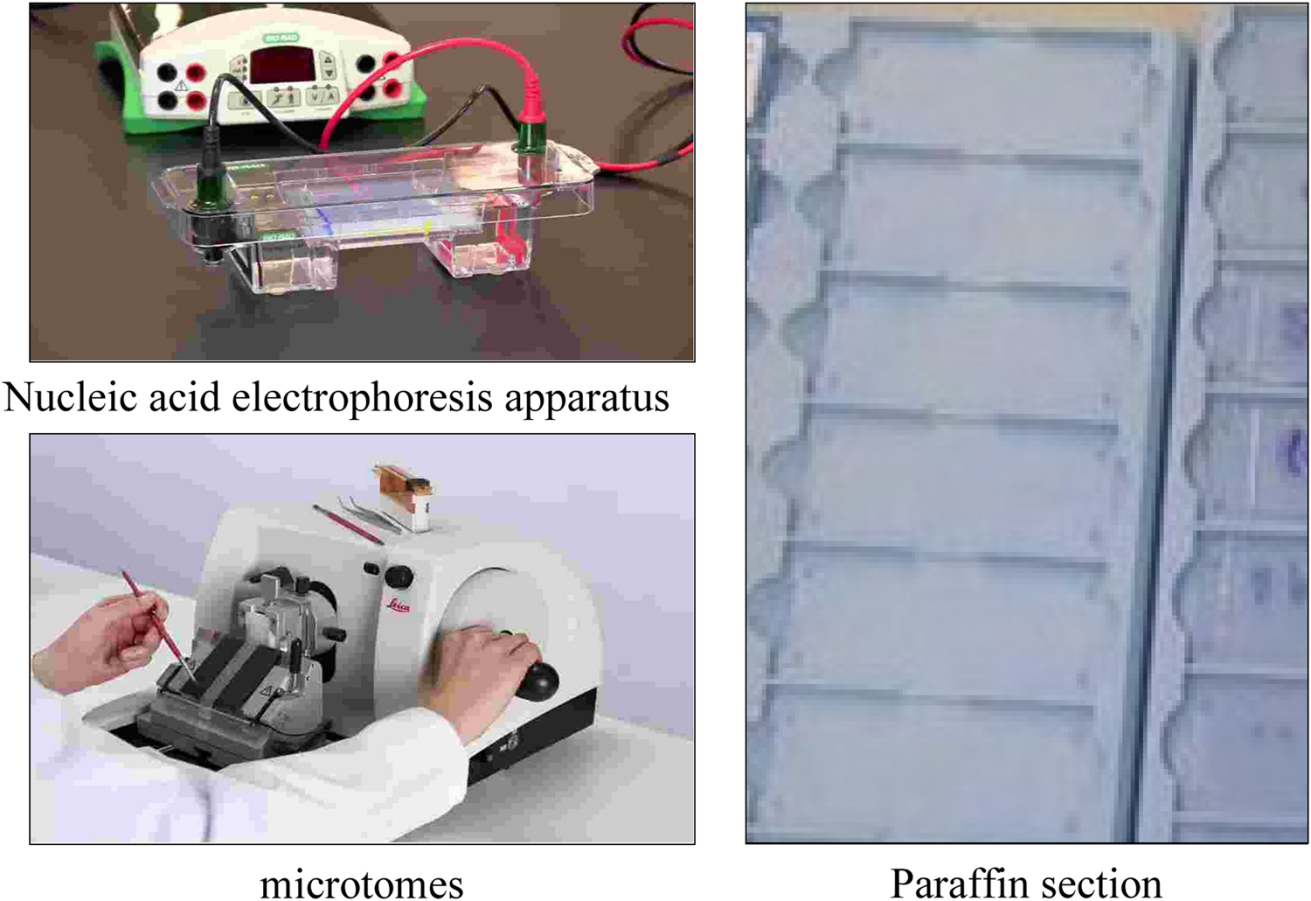
Parts of the experimental equipment.

The experimental instruments and reagents are shown in Tables 2 and 3, and some of the instruments are shown in Figure 6:

**Table 2:** Experimental equipment

Instrument	Factory
Electric heating constant temperature water tank	Shanghai-Heng Science Instrument Co., Ltd
Thermostatic metal bath	Hangzhou Bioer Technology Co., Ltd
Refrigerated high-speed centrifuge	BECKMAN
NanoVue	GE
Micro-wave oven	Galanz
PCR amplifier	Biotech Biotech
Nucleic acid electrophoresis instrument	Bio-Rad Corporation
Gel imager	Bio-Rad Corporation

**Table 3:** Experimental reagents

Reagent	Factory
Xylene, absolute ethanol	Beijing Chemical Plant
Phenol/chloroform/isopropanol	Biotech Biotech
Guanidinium isothiocyanate	Biotech Biotech
6xLoading buffer	Dalian Takara Bioengineering Company
DNA marker	Dalian Takara Bioengineering Company
Primer	Shanghai Sangon Bioengineering Co., Ltd
2xEasy Taq PCR Super Mix	Beijing Full Gold
Agarose Gel DNA Recovery Kit	AXYGEN

The extraction steps of genomic DNA in paraffin section lung cancer tissue are as follows. (1) Deparaffinization: it prepares 2–3 slices of paraffin tissue Section (5 μm), after removing excess paraffin around the tissue. The tissue section was put into a 1.5 mL Eppendorf tube, 1 mL of toluene was added, fully shaken, 56°C water bath for 15 min, inverted and mixed every 5 min, centrifuged at 12,000 rpm for 10 min, and the supernatant was removed. (2) Alcohol washing: adding 1 mL of absolute ethanol, mix well, centrifuge at 4°C for 5 min, and remove the supernatant. (3) Repeating dewaxing and alcohol washing once, and the steps are the same as above. (4) After the last alcohol wash, centrifuge at 4°C for 5 min, discard the supernatant, and dry at 37°C (20–30 min) until the pellet is completely dry. (5) After grinding the tissue with liquid nitrogen, transfer the tissue to a 2 mL EP tube. It was added with 1 mL of solution D and an equal volume of phenol/chloroform, vortexed for 10 s, and kept on ice for 10 min. Centrifuging at 12,000 rpm for 420 min, and transferring the supernatant (1,000 μL) to a new tube. (6) Adding an equal volume of cold isopropanol (800 μL), mixing well, and placed in a −20°C refrigerator overnight. (7) At 12,000 rpm for 410 min°C, discard the supernatant, washing twice with cold 75% ethanol, 12,000 rpm for 410 min°C, discarding the supernatant, and fully drying the DNA. (8) Resuspending with 30 μL sterilized triple-distilled water for later use.

### 4.3 Judgment of experimental results

The sequenced sequences were analyzed by DNAssitant software, and the sequencing results were compared with the EGFR and KRAS gene sequences in the gene bank with manual proofreading. If they were inconsistent, it was a mutation. Positive control: knowing positive specimen sections provided by the reagent company; negative control: using PBS instead of primary antibody as blank control. Expression location: positive staining for RRM1 protein indicated that brown-yellow granules of varying thickness were located in the cytoplasm, and positive staining for ERCC-1 protein indicated that the brown-yellow granules were located in the nucleus. Observation method: two or more attending pathologists observed the slices in a double-blind manner to determine the protein expression results. Positive judgment criteria: the staining intensity of cancer cells in the section: 0 points for no coloration of cells, 1 point for pale yellow, 2 points for brownish yellow, 3 points for tan (the staining depth should be compared with the background coloration). The number of positive cancer cells in the section is the percentage. It multiplied the positive staining intensity score by the percentage of positive tumor cells to obtain the final immunohistochemical score *H* value, which ranged from 0 to 300. The final median *H* value was used as the boundary, and the median *H* value greater than or equal to the median *H* value was judged as positive, and the median *H* value less than the median *H* value was judged as negative.

This article uses SPSS19.0 statistical software for analysis. The chi-square test was used to analyze the correlation, the Kaplan–Meier method was used to draw the disease-free survival curve, and the log-rank test was used between different stratification factors. Univariate and multivariate analyses were performed using COX proportional hazards regression model. Univariate analysis of statistically significant covariates was introduced into a multivariate COX proportional hazards regression model.

## 5 Curative effect prediction results

### 5.1 Overall characteristics of enrolled patients

**Figure 7:**
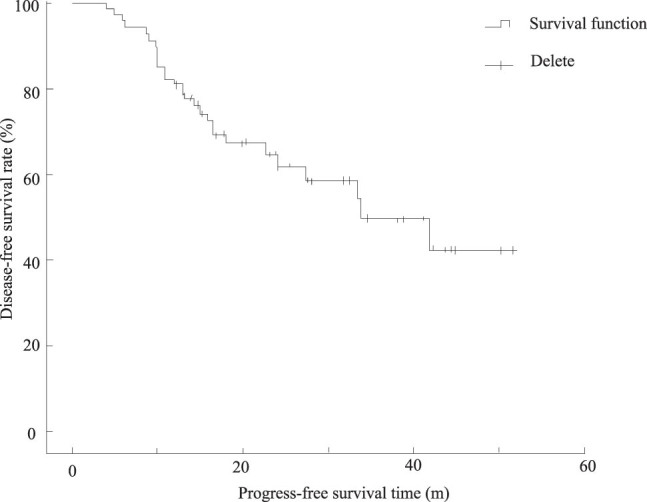
Disease-free survival curve of all patients (m).

In this study, March 1, 2013 was the cut-off date for follow-up, the follow-up rate was 95.6%, and the follow-up time was 9.1–51.8 months. The median follow-up time was 22.4 months. By the end of follow-up, 12 of 68 patients died, including 1 in stage I, 6 in stage II, and 5 in stage III. Recurrence or metastasis occurred in 27 cases, of which 12 cases recurred within 1 year after operation, and 10 cases recurred within 2 years after operation. The median recurrence-free survival time of all patients was 33.7 months, the 1-year disease-free survival rate was 80.9%, and the 2-year disease-free survival rate was 80.8%. The disease-free survival curves of all patients are shown in Figure 7.

### 5.2 EGFR mutation and postoperative adjuvant chemotherapy in NSCLC

**Figure 8:**
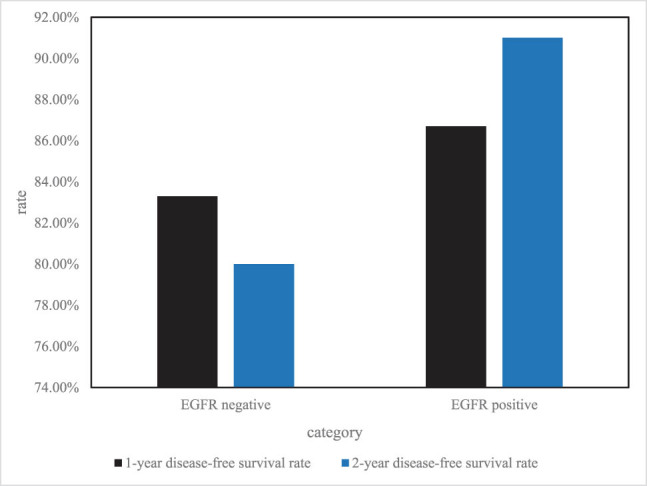
Comparison of disease-free survival in patients with different EGFR mutation status.

In the EGFR mutation-positive group, 14 of the 32 patients relapsed, whereas 13 of the 36 patients in the EGFR-negative group relapsed. The EGFR mutation-positive group had greater 1-year and 2-year disease-free survival rates than the EGFR mutation-negative group. The median time to progression was 33.7 months in the EGFR-positive group, which was not observed in the negative group. The *P* value for the comparison between the two groups was 0.519. The difference was not statistically significant, as shown in Figure 8.

**Figure 9:**
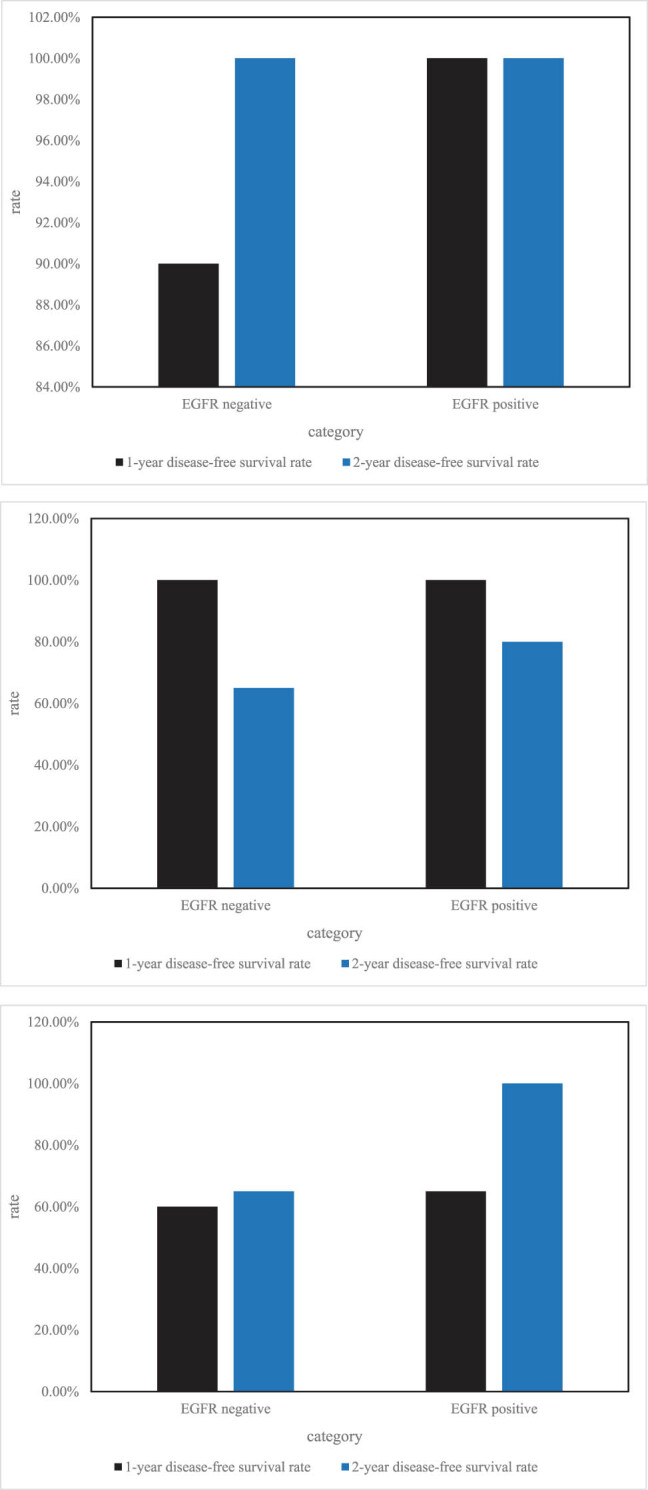
EGFR mutation status in different clinical stages.

According to different clinical stages, the disease-free survival rate of EGFR mutation status is quite different, as shown in Figure 9.

## 6 Conclusion

Based on the above evidence-based medical evidence, four courses of postoperative platinum-based adjuvant chemotherapy have become the standard treatment mode for early-stage NSCLC, but the treatment effect has entered a plateau. The 5-year absolute survival benefit is only 5%, which shows that most patients do not benefit from postoperative adjuvant chemotherapy. At present, there are few individualized studies led by biomarkers such as tumor driver genes and drug resistance proteins in postoperative adjuvant chemotherapy for early-stage NSCLC. This study included 68 patients with NSCLC who received GP regimen adjuvant chemotherapy after radical lung cancer resection. It detects the mutation of tumor driver genes EGFR and KRAS and the expression of drug resistance proteins ERCC1 and RRM1, respectively. Combined with clinical follow-up results, this article retrospectively analyzed the disease-free survival rate and median progression-free survival time of different gene mutation status and protein expression status, so as to provide the basis for individualized treatment of postoperative adjuvant chemotherapy for NSCLC. In the future, it can be expected to collect more comprehensive and accurate patient genome information, including the polymorphism of the CYP1B1 gene, and construct a more comprehensive integrated healthcare management through more precise gene sequencing technology. At the same time, advanced technologies such as artificial intelligence are utilized to conduct deep learning and mining on a large amount of data, constructing more accurate prediction models, and providing strong support for doctors to achieve more accurate personalized treatment.
